# Effect of intravitreal ranibizumab and aflibercept injections on retinal nerve fiber layer thickness

**DOI:** 10.1038/s41598-021-84648-1

**Published:** 2021-03-03

**Authors:** Jayoung Ahn, Kyuhwan Jang, Joonhong Sohn, Ji In Park, Daniel Duck-Jin Hwang

**Affiliations:** 1 Department of Ophthalmology, Hangil Eye Hospital, #35 Bupyeong-daero, Bupyeong-gu, Incheon, 21388 Korea; 2 Department of Medicine, Kangwon National University Hospital, Kangwon National University School of Medicine, Chuncheon, Gangwon-do Korea; 3 Department of Ophthalmology, Catholic Kwandong University College of Medicine, Incheon, Korea

**Keywords:** Eye diseases, Macular degeneration

## Abstract

The purpose is to evaluate the effects of multiple intravitreal ranibizumab (IVR) and aflibercept (IVA) injections on peripapillary retinal nerve fiber layer (RNFL) thickness in patients with exudative age-related macular degeneration (AMD). This retrospective, observational, consecutive case series study enrolled patients newly diagnosed with monocular exudative AMD from January 2014 to October 2019 who were administered IVR or IVA injections. Normal fellow eyes were included as controls. Medical records and spectral domain optical coherence tomography results were reviewed at baseline and at 3, 6, and 12 months after injection. No statistically significant differences in peripapillary RNFL thickness and intraocular pressure were observed between the treated and fellow eyes in the two groups. The global RNFL thicknesses for the treated eyes decreased significantly after 12 months compared with baseline, but no significant difference was observed in any of the six examined sectors (temporal, superior temporal, superior nasal, nasal, inferior nasal, and inferior temporal). At 12 months, the central macular thickness of the treated eyes decreased significantly. Multiple IVR and IVA injections are apparently safe considering peripapillary RNFL damage in patients with exudative AMD. The decreased RNFL thickness of the global sector was presumably due to anatomical improvement of macular lesions.

## Introduction

Exudative age-related macular degeneration (AMD) is a major cause of blindness^1,2^; however, the prognosis of patients with AMD has significantly improved since the development of intravitreal anti-vascular endothelial growth factor (VEGF) injections^3–5^. The use of anti-VEGF therapy has increased rapidly worldwide, raising a new concern—the potential for retinal nerve fiber layer (RNFL) damage owing to repeated anti-VEGF therapy.

RNFL damage can occur through two mechanisms. First, intraocular pressure (IOP) fluctuation owing to repeated intravitreal anti-VEGF injections can damage the RNFL^6–8^. However, previous studies have indicated that after an initial rapid rise in IOP owing to an increased intraocular volume following intravitreal injection, the IOP returns to the normal range within 10–30 min^9–11^. Secondly, VEGF is a neurotrophic factor; thus, suppression of the neuroprotective effects of VEGF with repeated anti-VEGF therapy can cause RNFL damage^12–15^.

Among the available anti-VEGF drugs, ranibizumab inhibits only VEGF-A, whereas aflibercept also binds to VEGF-B and to the placental growth factor^16^. Aflibercept also has a longer half-life and higher affinity for VEGF than ranibizumab^16–19^. Thus, aflibercept can theoretically cause more damage to the RNFL through greater VEGF suppression than ranibizumab. Although several studies have reported changes in RNFL thickness due to anti-VEGF therapy, there have been no studies on the effect of aflibercept injection on RNFL thickness^8,20–24^. Therefore, we evaluated the effects of multiple intravitreal ranibizumab (IVR) or aflibercept (IVA) injections on peripapillary RNFL thickness in patients with exudative AMD based on spectral domain optical coherence tomography (SD-OCT) measurements.

## Results

Patient demographics are shown in Table 1. The mean age of patients did not differ between the IVR and IVA groups. The other baseline characteristics, including sex, systemic disease (hypertension and diabetes), and number of injections, were also not significantly different between the IVR and IVA groups. There was no significant difference in baseline refractive error between the IVR and IVA group, and neither group showed any difference between the treated and fellow eyes.

**Table 1:** Baseline characteristics of all patients who received intravitreal injections.

	IVR	IVA	*p* value
No. of patients	29	29	
Age (years)	70.86 ± 8.56	73.00 ± 9.10	0.246^a^
Sex (male/female)	21/8	18/11	0.401^b^
**Systemic disease**			
Hypertension (n)	12	14	0.597^b^
Diabetes (n)	3	2	1.000^c^
Treated eye laterality (OD/OS)	17/12	13/16	0.293^b^
No. of injections	4.93 ± 1.39	4.69 ± 1.31	0.518^a^
**Refractive error (SE)**			
Treated eye	0.52 ± 1.41	0.47 ± 1.30	0.586^a^
Fellow eye	0.33 ± 1.23	0.30 ± 1.06	0.624^a^
*p* value (Treated vs. fellow)	0.436^a^	0.618^a^	

OD, right eye; OS, left eye; SE, spherical equivalent; IVR, group that received intravitreal ranibizumab injection; IVA, group that received intravitreal aflibercept injection.

^a^*p* value derived from Mann–Whitney *U*-test.

^b^*p* value derived from Pearson’s Chi-square test.

^c^*p* value derived from Fisher’s Exact test.

### Changes in BCVA and IOP

**Figure 1:**
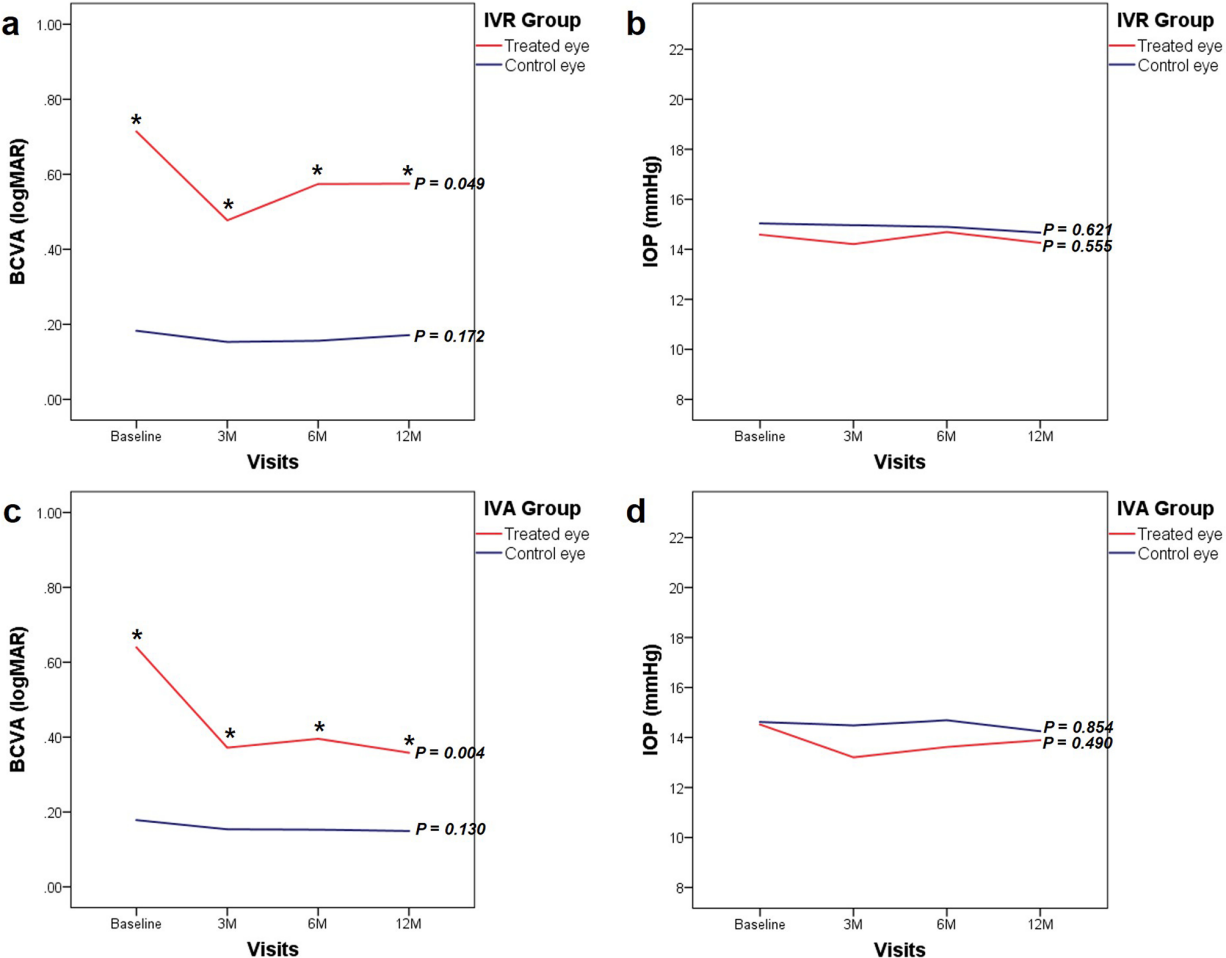
Changes in the best corrected visual acuity (BCVA) and intraocular pressure (IOP). (**a**) BCVA in the group administered intravitreal ranibizumab (IVR) injection. (**b**) IOP in the group administered IVR injection. (**c**) BCVA in the group administered intravitreal aflibercept (IVA) injection. (**d**) IOP in the group administered IVA injection. *p* Values (Wilcoxon signed-rank test) are shown for the differences between the baseline values and values at 12 months. Statistically significant differences (*p* < 0.05) between the values for the treated and control eyes are represented with asterisks (Mann–Whitney *U*-test).

In the IVR and IVA groups, the logMAR best corrected visual acuity (BCVA) of the treated eyes at 12 months (0.57 ± 0.62 and 0.35 ± 0.38, respectively; Snellen equivalent 20/74 ± 20/83 and 20/44 ± 20/47, respectively) significantly increased compared with the baseline values (0.71 ± 0.55 and 0.64 ± 0.54, respectively; Snellen equivalent 20/102 ± 20/70 and 20/87 ± 20/69, respectively). No significant change in visual acuity was observed in the fellow eyes. The BCVA of the fellow eyes was significantly higher than that of the treated eyes at all visits (Fig. 1).

In both groups, no significant change in IOP was observed at 12 months in the treated and fellow eyes and no significant difference was observed between the treated and fellow eyes at any visit (Fig. 1).

In a total of 143 injections in the treated eyes of the IVR group, the mean pre-injection IOP was 14.76 ± 2.57, and 1 day post-injection IOP was 14.65 ± 2.59. In a total of 136 injections in the treated eyes of the IVA group, the mean pre-injection IOP was 13.73 ± 3.27, and 1 day post-injection IOP was 13.61 ± 3.49. In both the groups, no significant change in IOP was observed 1 day after the injection (*p* = 0.318 and 0.477, respectively).

### Change in peripapillary RNFL thickness in the IVR group

The RNFL thicknesses of the global, superior temporal, temporal, inferior temporal, inferior nasal, nasal, and superior nasal sectors were not significantly different between the treated and fellow eyes in the IVR group at any visit (Table 2). However, the global RNFL thicknesses for the treated eyes significantly decreased at 3, 6, and 12 months compared with those at baseline (*p* = 0.002, 0.025, and 0.038, respectively). No significant differences were observed among the six sectors. The fellow eyes did not show any significant difference in the RNFL thickness at 12 months after treatment in all the sectors. No significant correlation was observed between the number of injections and the global RNFL thicknesses change (*p* = 0.234 derived from the Spearman correlation analysis).

**Table 2:** Variance in the thickness of retinal nerve fiber layer (μm) in the group administered intravitreal ranibizumab injection.

	Treated eye	Fellow eye	*p* value^a^
**Global RNFL**			
Baseline	101.03 ± 15.07	98.90 ± 14.33	0.608
Third month	99.24 ± 15.54	98.89 ± 15.34	0.967
Sixth month	98.14 ± 16.29	97.52 ± 16.53	0.973
Twelfth month	99.32 ± 14.07	98.00 ± 16.16	0.967
p value^b^	0.038*	0.812	
**Superior temporal sector RNFL**			
Baseline	133.86 ± 25.82	133.76 ± 30.54	0.785
Third month	134.83 ± 29.89	134.70 ± 32.30	0.812
Sixth month	138.21 ± 30.28	133.15 ± 34.58	0.637
Twelfth month	135.60 ± 26.48	134.61 ± 34.46	0.710
p value^b^	0.344	0.845	
**Temporal sector RNFL**			
Baseline	77.76 ± 18.80	71.41 ± 14.40	0.135
Third month	73.45 ± 17.12	72.00 ± 14.93	0.634
Sixth month	73.32 ± 16.56	70.89 ± 14.34	0.755
Twelfth month	74.24 ± 18.32	71.35 ± 15.16	0.556
*p* value^b^	0.058	0.614	
**Inferior temporal sector RNFL**			
Baseline	150.21 ± 26.55	145.48 ± 26.35	0.549
Third month	147.21 ± 26.72	147.93 ± 27.77	0.844
Sixth month	146.64 ± 24.92	147.63 ± 28.82	0.698
Twelfth month	149.96 ± 23.51	144.87 ± 28.32	0.650
*p* value^b^	0.435	0.476	
**Inferior nasal sector RNFL**			
Baseline	111.79 ± 21.98	110.69 ± 21.88	0.797
Third month	110.90 ± 22.58	110.19 ± 24.09	0.793
Sixth month	108.82 ± 22.37	110.07 ± 22.98	0.794
Twelfth month	109.72 ± 20.95	110.39 ± 23.04	0.975
*p* value^b^	0.939	0.489	
**Nasal sector RNFL**			
Baseline	70.69 ± 15.82	71.17 ± 12.16	0.613
Third month	70.45 ± 16.34	69.56 ± 12.11	0.967
Sixth month	71.32 ± 15.25	68.00 ± 13.90	0.464
Twelfth month	68.96 ± 15.76	68.83 ± 12.15	0.680
*p* value^b^	0.753	0.476	
**Superior nasal sector RNFL**			
Baseline	115.17 ± 22.15	116.07 ± 28.01	0.635
Third month	114.34 ± 21.97	114.26 ± 28.79	0.799
Sixth month	114.21 ± 21.25	113.52 ± 30.42	0.973
Twelfth month	112.96 ± 22.69	112.22 ± 30.82	0.926
*p* value^b^	0.123	0.305	

*RNFL* retinal nerve fiber layer.

^a^Comparison between the treated and fellow eyes in each period (Mann–Whitney *U*-test).

^b^Comparison between the baseline and twelfth month for each value (Wilcoxon signed-rank test).

* *p* <.05.

### Change in the peripapillary RNFL thickness in the IVA group

There was no significant difference in the RNFL thickness of any sector between the treated and fellow eyes at any visit among patients in the IVA group (Table 3). The global RNFL thicknesses for the treated eyes significantly decreased at 3 and 12 months compared with those at baseline (*p* = 0.033 and 0.023, respectively). No significant difference was observed in any of the six sectors. The fellow eyes showed no significant difference in the peripapillary RNFL thicknesses among all sectors at 12 months compared with those measured at the baseline examination. No significant correlation was observed between the number of injections and the global RNFL thicknesses change (*p* = 0.329 derived from the Spearman correlation analysis).

**Table 3:** Variance in the thickness of retinal nerve fiber layer (μm) in the group administered intravitreal aflibercept injection.

	Treated eye	Fellow eye	*p* value^a^
**Global RNFL**			
Baseline	97.39 ± 18.61	94.33 ± 17.96	0.281
Third month	95.43 ± 18.04	92.83 ± 18.56	0.354
Sixth month	96.75 ± 15.03	95.04 ± 14.54	0.399
Twelfth month	94.54 ± 17.71	93.58 ± 18.84	0.755
*p* value^b^	0.023*	0.831	
**Superior temporal sector RNFL**			
Baseline	128.07 ± 32.63	125.74 ± 30.86	0.459
Third month	126.11 ± 33.18	122.38 ± 30.62	0.317
Sixth month	129.96 ± 23.90	124.91 ± 20.00	0.208
Twelfth month	125.54 ± 32.76	122.85 ± 28.64	0.336
*p* value^b^	0.406	0.578	
**Temporal sector RNFL**			
Baseline	79.89 ± 20.71	75.19 ± 16.42	0.533
Third month	73.64 ± 16.86	72.33 ± 17.28	0.993
Sixth month	72.86 ± 15.75	74.00 ± 15.20	0.762
Twelfth month	74.39 ± 18.08	73.62 ± 16.42	0.952
*p* value^b^	0.134	0.870	
**Inferior temporal sector RNFL**			
Baseline	135.57 ± 38.87	137.04 ± 36.83	0.637
Third month	135.07 ± 38.58	134.75 ± 38.22	0.526
Sixth month	140.54 ± 30.38	141.00 ± 32.21	0.740
Twelfth month	132.32 ± 38.48	135.46 ± 41.02	0.856
*p* value^b^	0.057	0.394	
**Inferior nasal sector RNFL**			
Baseline	109.50 ± 31.15	104.44 ± 29.86	0.474
Third month	112.61 ± 28.50	105.25 ± 32.15	0.321
Sixth month	111.50 ± 23.79	106.43 ± 24.69	0.684
Twelfth month	108.68 ± 28.11	105.88 ± 29.93	0.835
*p* value^b^	0.932	0.445	
**Nasal sector RNFL**			
Baseline	69.89 ± 16.66	67.30 ± 12.17	0.292
Third month	69.82 ± 15.49	67.29 ± 11.44	0.195
Sixth month	70.18 ± 14.28	68.35 ± 12.75	0.495
Twelfth month	69.00 ± 14.45	66.73 ± 13.85	0.420
*p* value^b^	0.450	0.700	
**Superior nasal sector RNFL**			
Baseline	105.11 ± 29.90	102.33 ± 24.58	0.414
Third month	102.82 ± 29.62	100.33 ± 24.59	0.563
Sixth month	106.14 ± 25.31	103.70 ± 23.01	0.576
Twelfth month	102.64 ± 30.12	102.92 ± 24.54	0.869
*p* value^b^	0.100	0.779	

*RNFL* retinal nerve fiber layer.

^a^Comparison between the treated eyes and fellow eyes in each period (Mann–Whitney *U*-test).

^b^Comparison between the baseline and twelfth month for each value (Wilcoxon signed-rank test).

* *p* <.05.

### CMT in the IVR and IVA groups

In both groups, the CMT of the treated eyes was significantly higher than that of the fellow eyes at baseline and at 3 and 12 months after the injection (Table 4). In the IVR group, a significant difference was observed between the treated and fellow eyes at 3 months after the injection, but no significant differences were observed in the IVA group at the same time point (Table 4).

**Table 4:** Central macular thickness (μm) before and after intravitreal injection.

	Treated eye	Fellow eye	*p* value^a^
**IVR group**			
Baseline	450.55 ± 131.30	257.10 ± 27.94	< 0.001*
Third month	309.00 ± 81.06	265.68 ± 37.03	0.010*
Sixth month	338.66 ± 105.32	255.11 ± 28.35	< 0.001*
Twelfth month	333.42 ± 102.93	257.92 ± 35.34	0.004*
*p* value^b^	< 0.001*	0.848	
**IVA group**			
Baseline	436.21 ± 163.89	257.45 ± 24.42	< 0.001*
Third month	275.69 ± 74.47	257.28 ± 22.91	0.910
Sixth month	307.28 ± 92.59	257.64 ± 21.66	0.010*
Twelfth month	311.86 ± 118.29	255.20 ± 21.57	0.012*
*p* value^b^	< 0.001*	0.312	

IVR, group that received intravitreal ranibizumab injection; IVA, group that received intravitreal aflibercept injection.

^a^Comparison between the treated and fellow eyes during each period (Mann–Whitney *U*-test).

^b^Comparison between the baseline and twelfth month for each value (Wilcoxon signed-rank test).

* *p* <.05.

At 12 months, the CMT of the treated eyes significantly decreased compared with the baseline CMT, but no significant change in CMT was observed in the fellow eyes (Table 4).

## Discussion

To the best of our knowledge, this is the first study to evaluate the effects of IVA injection on the peripapillary RNFL. We included data on patients with treatment-naive exudative AMD in only one eye. Additionally, all the images were obtained using SD-OCT, which exhibits better reproducibility than time-domain OCT and provides the quantitative RNFL thickness at the global level and for the six divided sectors. We found no statistically significant differences in IOP or peripapillary RNFL thickness between the treated and fellow eyes at any visit in either the IVR or IVA group. In the comparisons between the values at baseline and12 months, a significant reduction in RNFL thickness was observed only in the global RNFL of the treated eyes in both groups.

In a study of 49 patients with AMD, Martinez-de-la-Casa et al.^8^ reported that the average RNFL of the treated eyes significantly decreased 12 months after ranibizumab injection with no significant difference in the control (fellow) eyes. This result is similar to that of the present study, although there was no comparison between the treated and fellow eyes in the previous study. However, other studies^20,25–28^ found no significant difference in the RNFL thickness after bevacizumab^20,25,26^ and ranibizumab^20,25–28^ injections. Martinez-de-la-Casa et al.^8^ reported that repeated intravitreal anti-VEGF injection caused RNFL thinning due to drug-related toxicity or IOP fluctuation. In contrast, Horsley et al.^20^ reported no episodes of sustained IOP increase in the eyes of 41 patients with exudative AMD in 27.0 ± 9.7 months, and concluded that repeated anti-VEGF injection does not adversely affect the RNFL thickness. Shin et al.^26^ reported that IOP fluctuation and the number of injections do not adversely affect the RNFL thickness in a study of patients with exudative AMD, diabetes mellitus retinopathy, and retinal vein occlusion. The researchers concluded that decreased RNFL thickness is associated with the severity of retinal ischemia. In the present study, no significant increase in IOP was observed 1 day after the injection, and no significant correlation was observed between the number of injections and the global RNFL thicknesses change.

The global RNFL thickness of the treated eyes in the IVR and IVA groups showed a significant decrease at 3 months (99.24 ± 15.54 and 95.43 ± 18.04 μm, respectively) compared with the baseline values (101.03 ± 15.07 and 97.39 ± 18.61 μm, respectively), and the values remained consistent at 12 months. Although the global RNFL thickness showed a significant decrease, it was within 3 μm, which is not clinically significant. We do not consider that the observed reduction in the global RNFL thickness of the treated eyes was an adverse effect of anti-VEGF injection. This reduction may instead be related to the anatomical improvement of macular exudative lesions after the initial three injections. Previous studies^20,25–28^ that reported no significant reduction in the RNFL thickness were not conducted in treatment-naive patients. Therefore, their macular lesions at baseline may be more stable than those in treatment-naive patients and have less effect on the peripapillary RNFL thickness. In our study and in the study of Martinez-de-la-Casa et al.^8^, which were both conducted in treatment-naive patients, the mean difference in the global RNFL thickness between treated and fellow eyes decreased from baseline to 12 months. In our study, the global RNFL thickness of the treated eyes decreased significantly at 12 months compared with that at baseline, whereas there was no significant difference from the fellow eyes at 12 months.

In addition, Jo et al.^23^ reported a decrease in the peripapillary RNFL thickness in the temporal quadrant and pathologic area owing to a macular lesion change in a study of patients with exudative AMD who received an IVR injection. Hwang et al.^29^ also reported that the peripapillary RNFL thickness was increased in patients with diabetic macular edema (DME), and the increment correlated with the degree of macular edema. They hypothesized that an increase in the RNFL thickness in the temporal sector in patients with acute DME might be related to the change in macular tomography due to macular edema. Because the nasal sector RNFL is less affected by macular lesion change, it can better reflect the damage caused by anti-VEGF injection. Martinez-de-la-Casa et al.^8^ reported no significant difference in the nasal quadrant RNFL thicknesses of the treated eyes at 12 months compared with those at baseline (*p* = 0.064*)*, whereas the temporal quadrant RNFL thicknesses were significantly decreased (*p* < 0.001*)*. In the present study, there was no significant decrease in the RNFL thickness of the temporal and nasal sectors in either group, although the temporal sector RNFL tended to decrease more than the nasal sector RNFL.

Although we observed greater macular anatomical improvement in the IVA group than in the IVR group, the peripapillary RNFL thickness results of the two groups were similar overall. In both the groups, only the global RNFL thickness of the treated eyes significantly decreased at 12 months. Furthermore, this was not a clinically significant change because of a minor variation. Therefore, we indirectly demonstrated that repeated aflibercept injection did not cause more RNFL damage in exudative AMD over 12 months than repeated ranibizumab injection in comparison of the treated and normal fellow eyes.

The first limitation of this study is its retrospective design. The lack of randomization might have resulted in baseline differences in disease severity between the groups. Therefore, we were unable to compare the two groups directly. However, as the findings can be used as a reference for future prospective randomized controlled studies, the comparison results of peripapillary RNFL thickness, CMT, BCVA and IOP between the IVR and IVA group are presented in Supplementary Tables S1 and S2. Secondly, we enrolled a relatively small number of patients. To overcome this limitation, we refined our study design. We enrolled typical patients with exudative AMD with only type I or type II choroidal neovascularization (CNV), excluding polypoidal choroidal vasculopathy (PCV) or retinal angiomatous proliferation (RAP), after thorough evaluation using multimodal imaging, including fluorescein angiography (FA) and indocyanine green angiography (ICGA). Finally, we could not standardize the injection intervals owing to the *pro re nata* protocol. Despite these limitations, this study provides valuable data on the effect of different anti-VEGF drugs in naive patients with AMD up to 1 year after intravitreal injection.

Importantly, no significant difference in the peripapillary RNFL thickness was observed between the treated and fellow eyes at any of the visits in both the groups. In conclusion, multiple IVR or aflibercept injections seem to be safe in terms of the potential for peripapillary RNFL damage in patients with exudative AMD. However, the RNFL thickness of the global sectors significantly decreased, presumably owing to the anatomical improvement of macular lesions. We believe that this study can help to alleviate the concern of physicians regarding RNFL damage after anti-VEGF injection.

## Methods

### Subjects

This was a retrospective, observational, consecutive case series. This study was performed in patients who were newly diagnosed with monocular exudative AMD at Hangil Eye Hospital (Incheon, Korea) from January 2014 to October 2019. Twenty-nine patients were administered intravitreal injections of ranibizumab (Lucentis, Novartis AG, Basel, Switzerland and Genentech, Inc., South San Francisco, CA, USA) and 29 patients were administered aflibercept (Eylea, Regeneron Pharmaceuticals, Tarrytown, NY, USA, and Bayer HealthCare, Berlin, Germany) by a single ophthalmologist (DDH). Medical records and SD-OCT (Spectralis OCT, Heidelberg Engineering, Heidelberg, Germany) results were reviewed retrospectively at baseline, and at 3, 6, and 12 months after injection.

The treatment group included eyes injected with IVR (0.5 mg/0.05 mL) or IVA (0.5 mg/0.05 mL). All the patients were treated with three injections at 1-month intervals after diagnosis, followed by additional *pro re nata* injections according to CNV activity. Normal fellow eyes were included as controls. The exclusion criteria were as follows: (1) a history of previous treatments that affect RNFL thickness, including vitrectomy, intravitreal injection, and intraocular laser treatment; (2) presence of other ocular diseases, such as retinal vascular disease, uveitis, glaucoma, and optic nerve disease; (3) diagnosis of exudative AMD in both eyes; and (4) diagnosis of PCV or RAP.

### Ophthalmic examinations

All patients underwent FA and ICGA at the baseline examination. Binocular BCVA and IOP were measured, and slit-lamp biomicroscopy, fundus photography, and SD-OCT were performed at each visit. SD-OCT was performed by a well-trained technician after pupil dilatation. Peripapillary RNFL thickness was measured by SD-OCT using the equipped software (Spectralis Nsite Axonal Analytics Software; Heidelberg Engineering, Heidelberg, Germany). The peripapillary RNFL thickness was measured in six sectors: temporal (315°–45°), superior temporal (45°–90°), superior nasal (90°–135°), nasal (135°–225°), inferior nasal (225°–270°), and inferior temporal (270°–315°). The global RNFL thickness was obtained by averaging the total 360° peripapillary RNFL thickness measurements. Tests in which the RNFL OCT quality did not satisfy the automatic real-time score of 16 or more and had a signal-to-noise ratio of 15 dB or more were excluded from the study. Macular thickness was measured by a volume scan of 30° centered on the fovea with a central fixation aid and 250 μm distance between scans. Central macular thickness (CMT) was confirmed by measuring the average thickness of the central 1-mm-diameter circle using the equipped software.

### Statistical analysis

Statistical analyses were performed using SPSS Statistics 23 software (SPSS Inc., Chicago, IL, USA). The results are expressed as mean ± standard deviation. The comparison between the treated and fellow eyes was conducted using Mann–Whitney *U*-test. The changes in values from the baseline examination to the 12-month follow up were analyzed using Wilcoxon signed-rank test. Results with a *p* value of less than 0.05 were considered statistically significant.

### Ethics approval

This study was performed in line with the principles of the Declaration of Helsinki. This study was approved by the institutional review board (IRB) of Hangil Eye Hospital (IRB number: 14004), and the requirement to obtain informed consent from study participants was waived by the IRB given the retrospective nature of the study.

## Supplementary Information

Supplementary Tables.

## Data Availability

The datasets generated during and/or analysed during the current study are available from the corresponding author on reasonable request.
