# Supplementary material for: Effect of intravitreal ranibizumab and aflibercept injections on retinal nerve fiber layer thickness
Source: Sci Rep. 2021 Mar 3;11:5010. doi: 10.1038/s41598-021-84648-1 (PMC7930121; doi:10.1038/s41598-021-84648-1)
Supplement: Supplementary file 1 — Supplementary Tables. [file 41598_2021_84648_MOESM1_ESM.docx]

**Effect of intravitreal ranibizumab and aflibercept injections on retinal nerve fiber layer thickness**

Jayoung Ahn^1^, Kyu Hwan Jang^1^, Joonhong Sohn^1^, Ji In Park^2^ & Daniel Duck-Jin Hwang^1,3,*^

^1^Department of Ophthalmology, Hangil Eye Hospital, Incheon, Korea; ^2^Department of Medicine, Kangwon National University Hospital, Kangwon National University School of Medicine, Chuncheon, Gangwon-do, Korea; ^3^Department of Ophthalmology, Catholic Kwandong University College of Medicine, Incheon, Korea

^*^Corresponding author: [daniel.dj.hwang@gmail.com](mailto:daniel.dj.hwang@gmail.com)

**Supplementary Table 1.** Comparison of retinal nerve fiber layer thickness (μm) of the treated eye between the intravitreal ranibizumab (IVR) and intravitreal aflibercept (IVA) groups.

|  | IVR | IVA | p value^a^ |
| --- | --- | --- | --- |
| Global RNFL |  |  |  |
| Baseline | 101.03 ± 15.07 | 97.39 ± 18.61 | 0.829 |
| Third month | 99.24 ± 15.54 | 95.43 ± 18.04 | 0.848 |
| Sixth month | 98.14 ± 16.29 | 96.75 ± 15.03 | 0.967 |
| Twelfth month | 99.32 ± 14.07 | 94.54 ± 17.71 | 0.782 |
| Superior temporal sector RNFL |  |  |  |
| Baseline | 133.86 ± 25.82 | 128.07 ± 32.63 | 0.655 |
| Third month | 134.83 ± 29.89 | 126.11 ± 33.18 | 0.555 |
| Sixth month | 138.21 ± 30.28 | 129.96 ± 23.90 | 0.231 |
| Twelfth month | 135.60 ± 26.48 | 125.54 ± 32.76 | 0.443 |
| Temporal sector RNFL |  |  |  |
| Baseline | 77.76 ± 18.80 | 79.89 ± 20.71 | 0.620 |
| Third month | 73.45 ± 17.12 | 73.64 ± 16.86 | 0.873 |
| Sixth month | 73.32 ± 16.56 | 72.86 ± 15.75 | 0.658 |
| Twelfth month | 74.24 ± 18.32 | 74.39 ± 18.08 | 0.635 |
| Inferior temporal sector RNFL |  |  |  |
| Baseline | 150.21 ± 26.55 | 135.57 ± 38.87 | 0.140 |
| Third month | 147.21 ± 26.72 | 135.07 ± 38.58 | 0.326 |
| Sixth month | 146.64 ± 24.92 | 140.54 ± 30.38 | 0.466 |
| Twelfth month | 149.96 ± 23.51 | 132.32 ± 38.48 | 0.127 |
| Inferior nasal sector RNFL |  |  |  |
| Baseline | 111.79 ± 21.98 | 109.50 ± 31.15 | 0.811 |
| Third month | 110.90 ± 22.58 | 112.61 ± 28.50 | 0.528 |
| Sixth month | 108.82 ± 22.37 | 111.50 ± 23.79 | 0.606 |
| Twelfth month | 109.72 ± 20.95 | 108.68 ± 28.11 | 0.943 |
| Nasal sector RNFL |  |  |  |
| Baseline | 70.69 ± 15.82 | 69.89 ± 16.66 | 0.672 |
| Third month | 70.45 ± 16.34 | 69.82 ± 15.49 | 0.690 |
| Sixth month | 71.32 ± 15.25 | 70.18 ± 14.28 | 0.506 |
| Twelfth month | 68.96 ± 15.76 | 69.00 ± 14.45 | 0.692 |
| Superior nasal sector RNFL |  |  |  |
| Baseline | 115.17 ± 22.15 | 105.11 ± 29.90 | 0.463 |
| Third month | 114.34 ± 21.97 | 102.82 ± 29.62 | 0.172 |
| Sixth month | 114.21 ± 21.25 | 106.14 ± 25.31 | 0.298 |
| Twelfth month | 112.96 ± 22.69 | 102.64 ± 30.12 | 0.289 |

^a^p value derived using Mann–Whitney *U*-test.; RNFL, retinal nerve fiber layer.

**Supplementary Table 2.** Comparison of the central macular thickness (CMT), best corrected visual acuity (BCVA), and intraocular pressure (IOP) of the treated eye between the intravitreal ranibizumab (IVR) and intravitreal aflibercept (IVA) groups.

|  | IVR | IVA | *P* value^a^ |
| --- | --- | --- | --- |
| CMT (μm) |  |  |  |
| Baseline | 450.55 ± 131.30 | 436.21 ± 163.89 | 0.465 |
| Third month | 309.00 ± 81.06 | 275.69 ± 74.47 | 0.054 |
| Sixth month | 338.66 ± 105.32 | 307.28 ± 92.59 | 0.205 |
| Twelfth month | 333.42 ± 102.93 | 311.86 ± 118.29 | 0.232 |
| BCVA (logMAR) |  |  |  |
| Baseline | 0.71 ± 0.55 | 0.64 ± 0.54 | 0.517 |
| Third month | 0.48 ± 0.54 | 0.37 ± 0.31 | 0.820 |
| Sixth month | 0.57 ± 0.63 | 0.40 ± 0.39 | 0.399 |
| Twelfth month | 0.57 ± 0.62 | 0.36 ± 0.38 | 0.213 |
| IOP (mmHg) |  |  |  |
| Baseline | 14.59 ± 2.96 | 14.52 ± 3.41 | 0.802 |
| Third month | 14.21 ± 2.97 | 13.21 ± 2.91 | 0.185 |
| Sixth month | 14.69 ± 3.00 | 13.62 ± 3.23 | 0.166 |
| Twelfth month | 14.26 ± 2.92 | 13.89 ± 3.22 | 0.456 |

^a^p value derived using Mann–Whitney *U*-test.
